# CRISPR–Cas12a for Birt–Hogg–Dubé syndrome: a promising diagnostic tool for deep intronic variant detection

**DOI:** 10.1097/MS9.0000000000004884

**Published:** 2026-03-30

**Authors:** Sakan Binte Imran

**Affiliations:** Department of Internal Medicine, Sir Salimullah Medical College, Dhaka, Bangladesh

*Dear Editor*,

Birt–Hogg–Dubé syndrome (BHD) is an autosomal dominant disorder characterized by cutaneous lesions, pulmonary cysts, spontaneous pneumothorax, and an increased risk of renal tumors. Although pathogenic variants in FLCN are responsible, a subset of clinically suspected patients remain genetically unresolved after standard testing. Conventional sequencing approaches primarily target coding exons and exon–intron boundaries and may fail to detect deep intronic variants^[^1^]^.

CRISPR-based diagnostic systems have emerged as highly sensitive tools for sequence-specific nucleic acid detection. Platforms utilizing Cas12a exploit collateral single-stranded DNase activity triggered upon target recognition, enabling amplification-coupled detection of single-nucleotide variants with high analytical sensitivity^[^2,3^]^. Such systems have demonstrated robust performance in identifying predefined mutations in infectious and genetic contexts^[^2,3^]^. This editorial aligns with the Transparency in the Reporting of Artificial Intelligence (TITAN) guideline, which emphasizes clarity and methodological transparency in emerging health technologies^[^4^]^.

In the setting of BHD, a targeted CRISPR–Cas12a assay could theoretically be designed to detect recurrent or suspected deep intronic FLCN variants that escape routine sequencing panels. Rather than replacing next-generation sequencing, this approach may serve as a complementary strategy in unresolved cases.

Previous work has demonstrated the feasibility of CRISPR-based approaches in detecting or correcting intronic mutations in other genetic diseases^[^5^]^. However, CRISPR–Cas12a diagnostics for deep intronic variants in BHD have not yet undergone clinical validation.

Important limitations must be addressed before clinical implementation. Accurate guide RNA design targeting deep intronic regions is technically demanding and requires rigorous validation^[^6^]^. Off-target cleavage and false-positive signals remain potential concerns and necessitate benchmarking against established sequencing methods in appropriately powered clinical cohorts^[^7^]^.

Prospective validation studies are essential to determine analytical sensitivity, specificity, reproducibility, and feasibility within routine diagnostic workflows. Demonstrating compliance with the World Health Organization’s ASSURED criteria would further support translational applicability^[^7^]^.

In conclusion, CRISPR–Cas12a represents a promising adjunctive strategy for detecting deep intronic variants in BHD. While clinical utility remains to be established, structured investigation may help close the diagnostic gap in unresolved cases.

## Data Availability

Not applicable.
